# Study on Adsorption and Aggregation in the Mixed System of Polyacrylamide, Cu(II) Ions and Innovative Carbon–Silica Composite

**DOI:** 10.3390/polym12040961

**Published:** 2020-04-20

**Authors:** Katarzyna Szewczuk-Karpisz, Viktor M. Bogatyrov, Mariia Galaburda, Zofia Sokołowska

**Affiliations:** 1 Institute of Agrophysics, Polish Academy of Sciences, Doświadczalna 4, 20-290 Lublin, Poland; sekretariat@ipan.lublin.pl; 2 O.O. Chuiko Institute of Surface Chemistry, National Academy of Sciences of Ukraine, General Naumov Street 17, 03164 Kiev, Ukraine; info@isc.gov.ua (V.M.B.); mariia.galaburda@gmail.com (M.G.)

**Keywords:** mixed adsorption layer, adsorption and aggregation mechanism, particle sizeanalysis, turbidimetry, flocculation

## Abstract

The paper presents an original study on adsorption and aggregation phenomena in a mixed system consisting of a macromolecular compound, heavy metal ions and an innovative adsorbent. The authors used ionic polyacrylamides (PAM), Cu(II) ions and carbon–silica composite (C-SiO_2_) in the experiments. Such a system has not yet been described in the literature and therefore, the article is of significant novelty and great importance. The composite was prepared by mixing phenol–formaldehyde resin with silica and pyrolysis at 800 °C. The adsorbed amounts of Cu(II) ions and PAM were determined spectrophotometrically. C-SiO_2_ was characterized using potentiometric titration, microelecrophoresis and Fourier Transform Infrared Spectroscopy (FTIR) analysis. In turn, the C-SiO_2_ aggregation was established turbidimetrically as well as using a particle size analyzer. The obtained results indicated that both Cu(II) ions and ionic polyacrylamide were adsorbed on the composite surface at pH 6. The highest noted adsorbed amounts were 9.8 mg/g for Cu(II) and 35.72 mg/g for CT PAM-25%. Cu(II) ions increased the anionic PAM adsorbed and reduced the cationic PAM one. The adsorption of anionic PAM (50 ppm) stimulated the solid aggregation significantly. What is more, Cu(II) ions enhanced this process. The size of particles/aggregates formed without additives equaled 0.44 μm, whereas in the mixed Cu(II)/AN PAM system, they were even at 1.04 μm.

## 1. Introduction

Adsorption is one of the main surface phenomena occurring in many physical, chemical and biological systems, both natural and artificial [1]. This process is commonly applied in industry and research. Adsorption is of great importance in implantology. It decides whether the implant will be accepted or rejected by the body [2]. This process is also significant in environmental protection. Various types of adsorbents are used to remove toxic substances from water and wastewater [3]. In a soil environment, adsorption leads to the immobilization of heavy metal ions and thereby reduces their availability to organisms [4]. In medicine and biotechnology, the described phenomenon is very helpful in the preparation of biosensors and drug delivery systems [5]. In the food industry, it facilitates the removal of various types of sediments from beverages [6]. What is more, adsorption is applied in various types of catalysts [7].

The adsorption efficiency is highly dependent on the type and properties of used adsorbent. Its textual properties are extremely important, i.e., specific surface area and porosity, particle size, as well as surface charge density. Scientists from many research centers are working on new adsorbents that allow for the effective removal of various substances. There are studies on the separation of many organic molecules such as surfactants, polymers, dyes and antibiotics from aqueous media. Some researchers used alumina particles modified with anionic surfactant in removal of oxytetracycline and heavy metals [8,9]. Others applied alumina of different particle size in poly(styrenesulfonate) adsorption [10]. Pham and co-workers synthesized nanosilica from rice husk and used it as an adsorbent of antibiotics, i.e., beta-lactam cefixime and ciprofloxacin [11,12,13]. The same team prepared modified adsorbents from laterite soil and used them in cationic dye removal [14]. Tomczyk et al. used biochar for copper(II) immobilization [15]. Adamczuk and Kołodyńska described the removal of heavy metals using modified fly ash [16,17]. Nowicki et al. prepared carbonaceous adsorbents from coffee waste materials and tropical fruit skins [18,19]. Orooji et al. synthesized a nanocomposite of silver iodide/graphitic carbon nitride as well as titania/polyether sulfone [20,21]. Irani-nezhad et al. described nanocomposite with peroxidase-like activity [22]. Hassandoost et al. developed heterojunctions based on Ce^3+^/Cr^4+^ modified Fe_3_O_4_ nanoparticles anchored onto graphene oxide [23]. Karimi-Maleh et al. focused on the application of magnetite/grapheme oxide nanocomposite [24]. In turn, Zhang et al. described porous magnetic carbon sheets from biomass [25].

Therefore, it may be stated that there are many papers describing adsorption of heavy metal ions or organic compounds on the various solids in the single systems, i.e., composed of one adsorbate type. But, this process occurring in the mixed systems is scarcely reported in the literature. Due to this fact, our team focused on this issue. Fijałkowska et al. [26] determined anionic polyacrylamide impact on lead(II) immobilization on kaolinite surface. The same authors [27] examined the effect of polyacrylamide containing quaternary amine groups on the Pb(II) and Cr(VI) accumulation on montmorillonite. Wiśniewska and Nowicki described simultaneous removal of organic molecules from a mixed system using peat-based activated carbons [28], as well as the adsorption of lead(II) and polyacrylic acid from a mixed solution using biocarbons prepared from corncob and peanut shell precursors [29]. In turn, Szewczuk-Karpisz et al. [30] focused on the adsorption capacity of hay-based activated biochars in the mixed system of Cu(II) ions and polyacrylamide.

In this paper, the effect of simultaneous adsorption of Cu(II) ions and ionic polyacrylamide (PAM) on the aggregation of carbon–silica composite (C-SiO_2_) was determined. In other words, the adsorptive and aggregation properties of innovative C-SiO_2_ adsorbent were examined in the mixed Cu(II)/PAM system. The solid was prepared by pyrolysis of the mixture (1:1) of phenol–formaldehyde resin and silica. The obtained composite was characterized using Fourier Transform Infrared Spectroscopy (FTIR) and nitrogen adsorption/desorption method. The adsorption/aggregation mechanism was proposed based on the adsorption study, potentiometric titration and zeta potential study results. The aggregate formation without and with adsorbates was established using a particle size analyzer and turbidimeter. The presented results are of high importance. They supplement the literature with information on adsorption and aggregation of solids in the mixed heavy metal/polymer systems. Moreover, the paper provides information on whether the selected solid can be used during water and wastewater treatment as an effective adsorbent of copper(II) and polyacrylamide. In addition, whether it can be applied as a soil additive to immobilize Cu(II) ions, even in the presence of soil polyacrylamide flocculant.

## 2. Materials and Methods

Carbon–silica composite, marked as C-SiO_2_, was used in the experiments as the adsorbent. It was prepared at Chuiko Institute of Surface Chemistry, National Academy of Sciences of Ukraine. At the beginning, pyrogenic silica A-300 (Pilot plant of Institute of Surface Chemistry NAN of Ukraine, Kalush, Ukraine) and phenol–formaldehyde resin of novolac type (JSC ‘Ukrainian resins’, Kalush, Ukraine) were grinded by a porcelain ball mill at a 1:1 ratio for 2 h. Then, the obtained mixture was pyrolyzed in a stainless steel reactor at 800 °C for 2 h in an argon flow. The composition of final product was determined thermogravimetrically and was as follows: 31.5% by weight carbon, 63.6% SiO_2_ and 4.9% adsorbed H_2_O. Textural properties of C-SiO_2_ were also measured using low-temperature nitrogen adsorption/desorption method (sorptometer ASAP 2020, Micrometrics). The specific surface area (S_BET_) of the solid was 297 m^2^/g, whereas the average pore diameter equaled 6.1 nm. It was found that the C-SiO_2_ composite was characterized by polydisperse porous structure consisting mainly of pores in the diameter ranges of 0.54–0.59 nm, 1.27–1.36 nm and 25.25–86.24 nm. 

**Figure 1:**
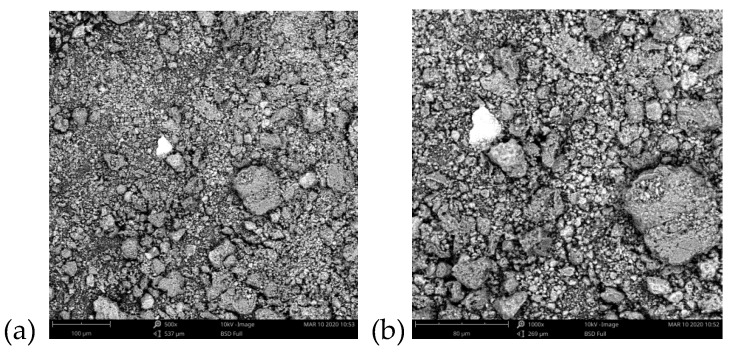
Scanning Electron Microscope (SEM) images of the C-SiO_2_ composite of magnification 500× (a) and 1000× (**b**).

The images of C-SiO_2_ were made using a scanning electron microscope, Phenom ProX (Thermo Fisher Instruments, Somerset, NJ, USA) with the magnification of 500 and 1000. They are presented in Figure 1.

Surface charge density (σ_0_) as a function of pH value as well as point of zero charge (pH_pzc_) of the solid was established using the potentiometric titration method and the software ‘titr_v3’ [31]. The apparatus consisted of a Teflon vessel thermostated using thermostat RE 204 (Lauda), glass and calomel electrodes (Beckman Instruments), pHmeter PHM 240 (Radiometer, Copenhagen, Denmark), microburette Dosimat 765 (Metrohm, Herisau, Switzerland) and computer. The σ_0_ parameter was calculated based on the difference in the base volume added to the C-SiO_2_ suspension and the supporting electrolyte solution (0.001 mol/dm^3^ NaCl) assuring the specific pH value. The examined systems were prepared by addition of 0.1 g of C-SiO_2_ to 50 cm^3^ of the supporting electrolyte solution. 0.1 mol/dm^3^ NaOH was used as a titrant. A single titration was performed in the pH range of 3–10. 

Electrophoretic mobility as a function of pH value as well as isoelectric point (pH_iep_) of the adsorbent were determined using the microelectrophoresis method (zetameter Nano ZS, Malvern Instruments, Worcestershire, UK). 0.02 g of the solid was added to 200 cm^3^ of the supporting electrolyte (0.001 mol/dm^3^ NaCl) solution. Such prepared suspension was divided into several parts (of different pH value in the range of 3–10). After 3-min sonication, the zeta potential was measured. Zeta potential (ζ) of the solid was calculated using Henry’s equation [32]. 

Surface groups of C-SiO_2_ were determined using Fourier-transform infrared spectroscopy (Nicolet 8700A FTIR spectrometer, Thermo Scientific, Somerset, NJ, USA). The solid was examined in pellet form with KBr.

Modified, ionic polyacrylamide (PAM), delivered by Korona JV, was used in the study as adsorbate. There were 4 types of PAM: (1) cationic PAM containing 15% of ionizable groups (CT PAM-15%), (2) cationic PAM containing 25% of ionizable groups (CT PAM-25%), (3) anionic PAM containing 12% of ionizable groups (AN PAM-12%) and (4) anionic PAM containing 40% of ionizable groups (AN PAM-40%). In the case of cationic PAMs, quaternary amine groups were able to ionize, whereas in the case of anionic PAMs—carboxylic ones. The average molecular weight ($\bar{M_{w}}$) of the polymers was: 12,000 kDa (AN PAM-12%), 13,000 kDa (AN PAM-40%), 7200 kDa (CT PAM-15%) and 6800 kDa (CT PAM-25%). The pK_a_ values of anionic polymers were: 4.4 (AN PAM-12%), 5.3 (AN PAM-40%. In turn, the pK_b_ values of cationic ones equaled: 9.4 (CT PAM-15%) and 9.6 (CT PAM-25%). At pH 6, dissociation degrees (α) of used polyacrylamides were equal to: 0.98 (AN PAM-12%), 0.83 (AN PAM-40%), 0.99 (CT PAM-15%) and 0.99 (CT PAM-25%). The above parameters were determined using potentiometric titration as well as the following equations [33]:

(1) $$pH-pK_{a}=log\frac{\propto }{1-\propto }$$

*pK_a_* + *pK_b_* = 14 (2)

**Figure 2:**
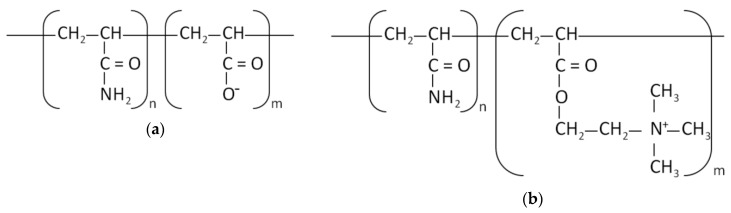
The monomer structure of anionic polyacrylamide (**a**) and cationic one (**b**).

The structure of the monomers of the used PAMs is presented in Figure 2.

Cu(II) ions were also used as adsorbate in this study. Their stock solution was prepared using CuCl_2_.

Adsorbed amounts of ionic polyacrylamide and copper(II) ions on the C-SiO_2_ surface were determined based on the reduction in their concentration in the solution after the adsorption process. The samples were prepared by addition of 0.02 g of C-SiO_2_ to 10 cm^3^ of the solution containing supporting electrolyte (0.001 mol/dm^3^ NaCl) and 50 or 100 ppm of PAM/Cu(II). When the pH value of the samples was adjusted to the value of 6, the adsorption was started and conducted by a specific time (24 h in the case of polyacrylamide adsorption, 1 h – Cu(II) adsorption), which were established based on the kinetics study. After the process completion, the samples were filtered and the concentration of adsorbate was determined spectrophotometrically (Jasco V-530 UV/Vis spectrophotometer) in the obtained clear solutions. The concentration of anionic polyacrylamide was determined using hyamine 1622 solution [34] 1.5 ml of this indicator was added to 5 ml of the examined sample and the obtained turbidity was measured after 5 min at λ = 500 nm. Cationic polyacrylamide concentration was determined using brilliant yellow. At the beginning, the pH value of the studied solutions was adjusted to the value of 9.5. Then, 0.5 ml of the sample was introduced to 4.5 ml of the indicator and the obtained orange-yellow color was measured at λ = 495 nm. Copper(II) ions concentration was determined using ammonia [35]. 50 µl of the indicator was added to 5 ml of the examined solution and the obtained dark-blue color was measured at λ = 620 nm. The Cu(II) ions effect on the PAM adsorbed amount as well as PAM impact on the copper(II) adsorption were measured in the samples containing 50 or 100 ppm of PAM and 100 ppm of Cu(II). 

Aggregation tendency of the C-SiO_2_ composite without and with Cu(II) ions and/or PAM was established turbidimetrically (turbidimeter Hach 2100AN, Omc Envag, Poland). The probes were prepared by addition 0.04 g of C-SiO_2_ to 20 cm^3^ of the solution containing supporting electrolyte (0.001 mol/dm^3^ NaCl). After a 3-min sonication, the polymer or heavy metal ions were added to the system and the pH value was adjusted to 6. In the next step, the measurement was started and the turbidity of the sample was registered after 2, 5, 10, 30, 60 and 90 min. 

Diameter of the particles/aggregates present in the C-SiO_2_ suspension without and with ionic polyacrylamide and/or copper(II) ions was determined using CPS analyzer (CPS Instruments, Anaheim, CA, USA). In the experiments 8% and 24% sucrose were used in the gradient formation. The disc rate was 2500 rpm, whereas the diameter range equals 0.12-12 µm. The samples were prepared in the same way as for turbidimetric analyzes. 

Each measurement was repeated three times. The measurement errors of zeta potential, potentiometric titration, turbidimetry and CPS analysis were very small and did not exceed 3% (thus the error bars were not visible on the graphs). In turn, the measurement error of adsorption study did not exceed 5%. 

## 3. Results

### 3.1. Surface Charge Density, Zeta Potential and Functional Groups of the Carbon–Silica Composite

**Figure 3:**
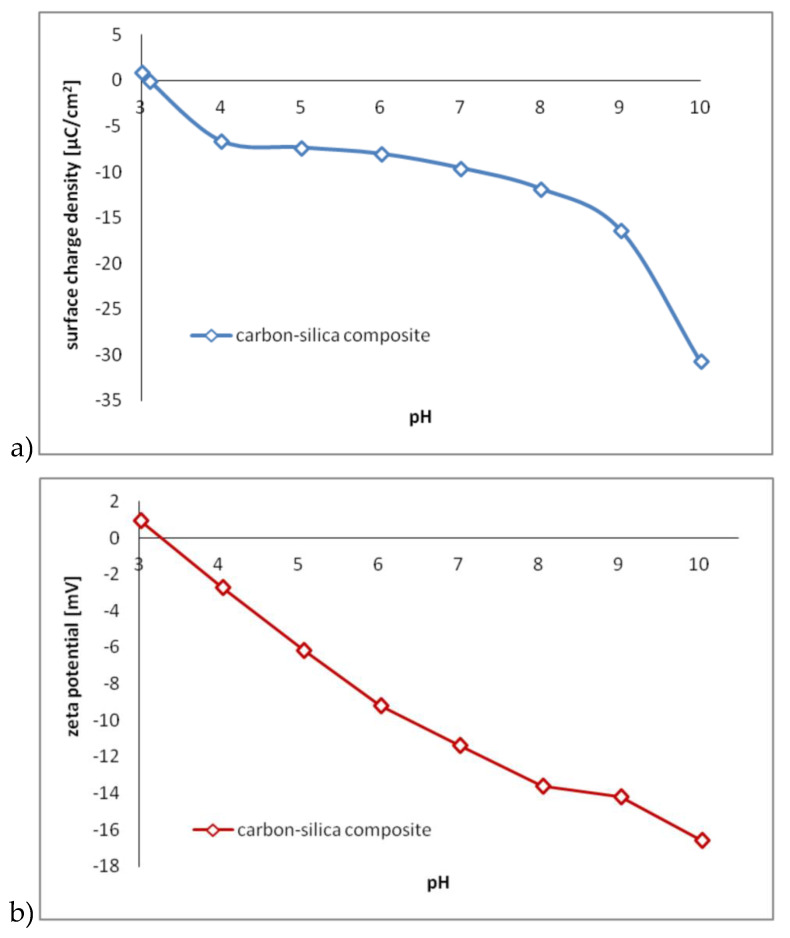
Surface charge density (**a**) and zeta potential (**b**) of the carbon–silica composite as a function of pH value.

The carbon–silica composite was characterized by determination of its surface charge density, zeta potential and surface functional groups. The dependency of C-SiO_2_ surface charge density and zeta potential on solution pH value is presented in Figure 3.

Potentiometric titration indicated that point of zero charge (pH_pzc_) of the carbon–silica composite was about 3.1. This means that at pH 3.1 the C-SiO_2_ surface is characterized by zero surface charge. The amounts of positive and negative surface groups are almost identical. At pH<3.1 the solid surface is positively charged, in turn at pH>3.1 – negatively charged. On the other hand, microelectrophoresis study showed that isoelectric point (pH_iep_) of selected composite was 3.2. Thus, at pH 3.2 the net charge of the C-SiO_2_ slipping plane is close to 0. At pH<3.2, positive groups dominate in this structure (zeta potential has positive values), whereas at pH>3.2—negative moieties prevail (zeta potential is negative). Analogous dependency of zeta potential and surface charge density on solution pH value was observed during various studies [36,37].

**Figure 4:**
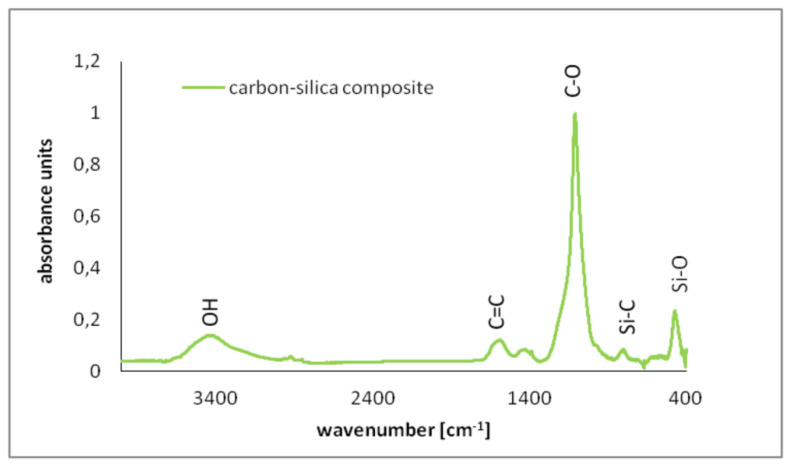
**Fourier Transform Infrared Spectroscopy** (FTIR) spectrum of the carbon–silica composite.

The obtained FTIR spectrum of C-SiO_2_ is presented in Figure 4.

The FTIR analysis indicated the specific groups on the C-SiO_2_ surface. In the obtained spectrum the following bands were noted: (1) 3374 cm^-1^ (attributed to OH groups stretching), (2) 1561 cm^-1^ (C=C stretching in aromatic rings), (3) 1381 cm^-1^ (O-H bonds deformation), 1096 cm^-1^ (C-O bonds stretching), 782 cm^-1^ (Si-C bonds stretching), 462 cm^-1^ (Si-O bonds stretching). Similar bonds were also observed and described by other researchers [38,39,40]. 

### 3.2. Adsorbed AMount of Ionic Polyacrylamide and Cu(II) Ions on the C-SiO_2_ Surface in the Single and Mixed Systems

**Figure 5:**
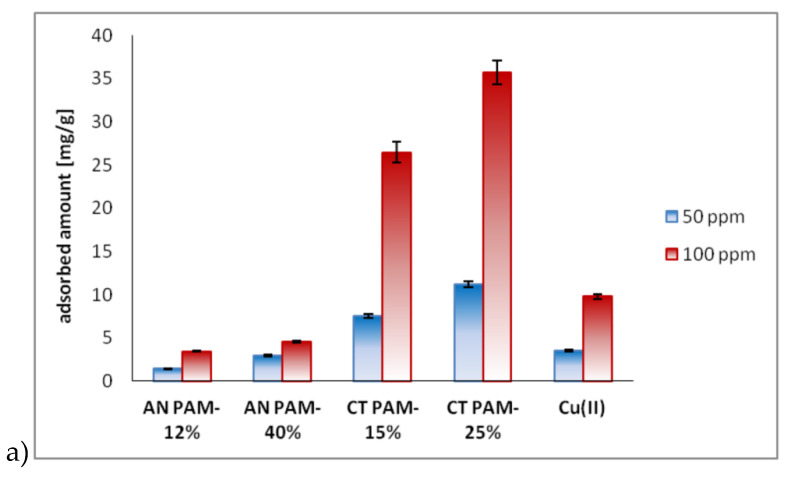

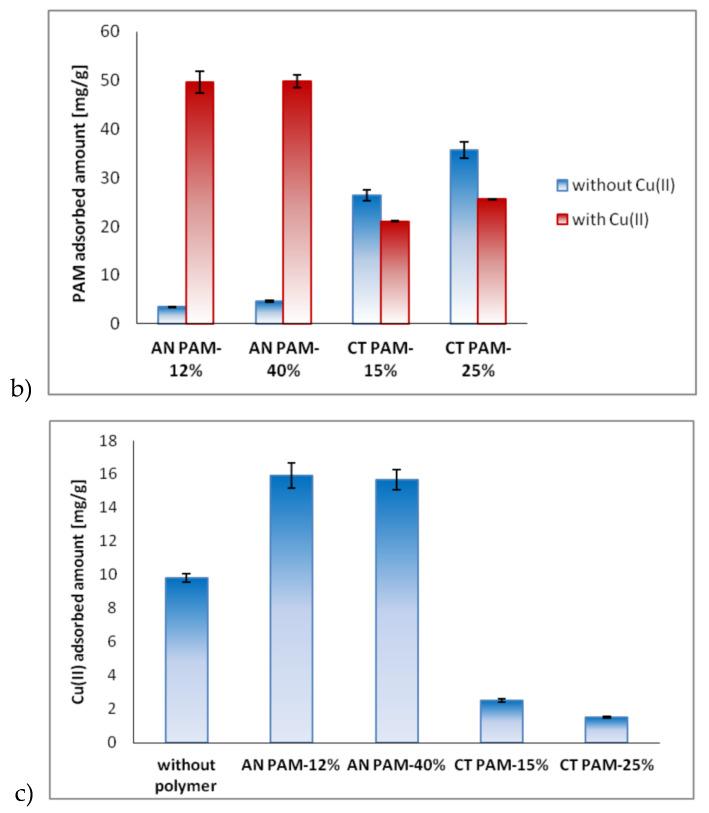
Ionic polyacrylamide and copper(II) ions adsorbed amounts on C-SiO_2_ in the single systems (the initial concentration of adsorbates was 50 or 100 ppm) (**a**) or mixed PAM/Cu(II) systems (the initial concentration of adsorbates was 100 ppm) (**b**,**c**) at pH 6.

The results of adsorption study performed in the single and mixed Cu(II)/PAM systems are presented in Figure 5. 

Figure 5a showed that the highest PAM adsorbed amounts on the C-SiO_2_ composite were noted for the polymer containing quaternary amine groups (CT PAM). The adsorbed amounts of anionic polyacrylamides (AN PAM) were significantly lower. For the initial PAM concentration 100 ppm, the measured adsorbed amounts were as follows: 3.45 mg/g for AN PAM-12% (6.9% of the ions were adsorbed), 4.62 mg/g (9.14%) for AN PAM-40%, 26.46 mg/g (52.92%) for CT PAM-15% and 35.72 mg/g (71.16%) for CT PAM-25%. What is more, the selected sorbent bonded a certain amount of Cu(II) ions. For the initial Cu(II) concentration 100 ppm, its adsorbed amount was 9.8 mg/g. Then the Cu(II) adsorption efficiency was 19.6%.

The PAM adsorbed amounts noted in the Cu(II) presence were different than those measured in the single system. As can be seen in Figure 5b, Cu(II) ions increased the adsorbed amounts of anionic polyacrylamide and reduced those of cationic polyacrylamide. When 100 ppm of Cu(II) ions were added to the system, the AN PAM-12% and AN PAM-40% adsorbed amounts were 49.68 mg/g and 49.83 mg/g, respectively. Then the adsorption efficiency reached almost 100%. The CT PAM adsorbed amounts in the Cu(II) ions presence were not so large. For CT PAM-15%, this parameter equaled 21.08 mg/g, while for CT PAM-25%—25.6 mg/g. The adsorption efficiency was 42.16% and 51.8%, respectively.

On the other hand, polyacrylamides also affected the Cu(II) ions adsorption on the C-SiO_2_ composite. AN PAM contributed to higher adsorption of heavy metal ions, whereas CT PAM made the Cu(II) adsorption lower (Figure 5c). The Cu(II) adsorbed amount in the AN PAM-12% or AN PAM-40% presence was about 16 mg/g (32% of the ions were adsorbed). In turn, in the CT PAM-15% and CT PAM-25% presence this parameter equaled only 2.5 mg/g (5%) and 1.5 mg/g (3%), respectively.

### 3.3. C-SiO_2_ Aggregation without and with Ionic Polyacrylamide/Cu(II) Ions in the Simple and Mixed Systems

**Figure 6:**
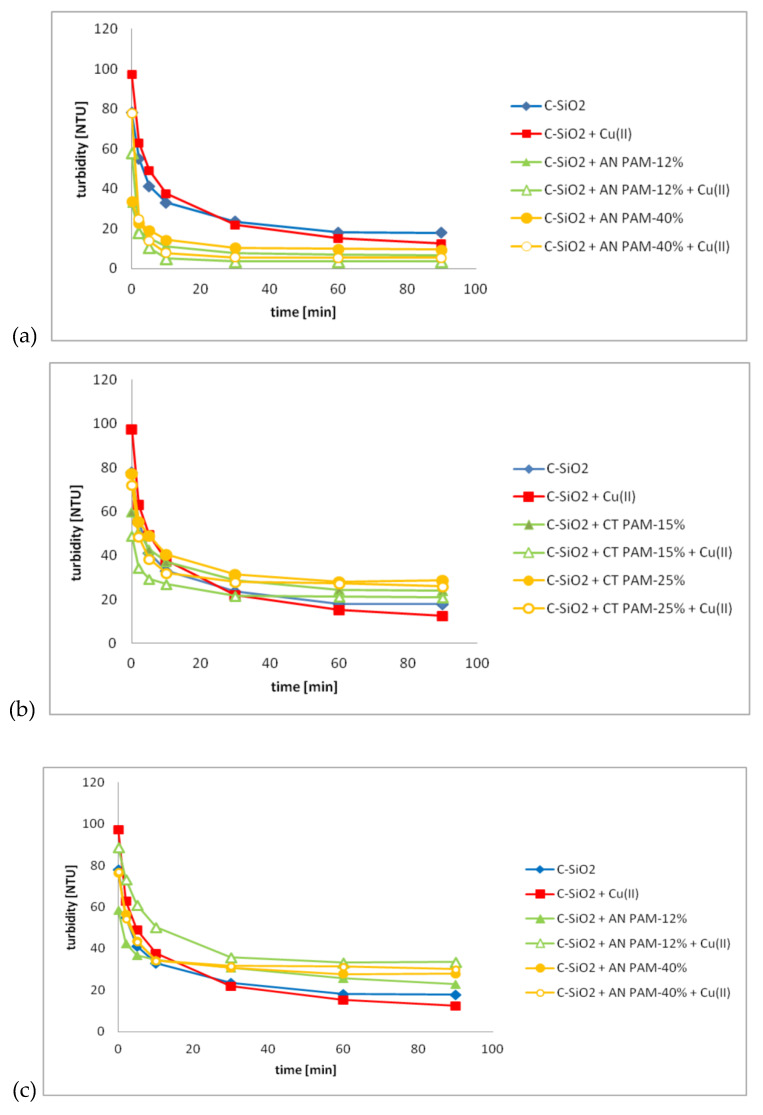

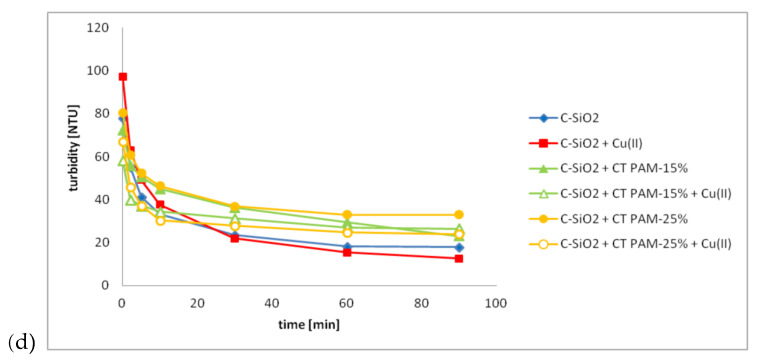
Turbidity changes of the C-SiO_2_ suspension without and with ionic polyacrylamide and/or Cu(II) ions over time at pH 6; the PAM concentration was 50 ppm (**a**,**b**) or 100 ppm (**c**,**d**), whereas the Cu(II) one—100 ppm.

The C-SiO_2_ aggregation tendency in the simple and mixed PAM/Cu(II) systems was established based on the results of turbidimetric measurements. They are presented in Figure 6.

The obtained results indicated that all adsorbates significantly influenced the C-SiO_2_ aggregation. When 50 ppm of AN PAM was added to the system, there was a fast formation of aggregates that fell into the vial bottom. As a consequence, the suspension was clear just after 10 min (the system turbidity did not exceed 20 NTU). The same concentration of CT PAM had different effect on the C-SiO_2_ aggregation. It was noted that some of the particles formed aggregates that fell into the vial bottom, but some of them remained suspended in the solution. As a result, the clarification of the system did not occur. The measured turbidity of the suspension was relatively high and equaled even 40 NTU after 10 min for the system containing 50 ppm of CT PAM-25%. Higher PAM concentration (100 ppm) had a similar effect. In the systems containing such amount of the polymer (AN PAM or CT PAM), the solid aggregation was not enhanced enough to observe a clear suspension. Some of the particles remained suspended in the system and thus the noted turbidity was relatively high.

The Cu(II) ions added to the system did not affect the turbidity of the C-SiO_2_ suspension significantly. In most cases, when the heavy metal ions and the polymer were added to the system at the same time, the aggregation tendency of the solid was similar to that observed in the suspension containing only the macromolecular compound.

**Figure 7:**
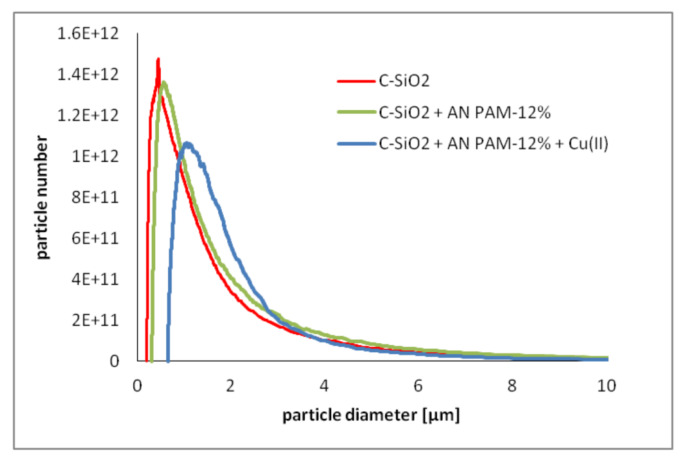
The particle number vs. particle diameter for the C-SiO_2_ suspension without and with AN PAM-12% and/or Cu(II) ions at pH 6; the PAM concentration was 50 ppm, whereas the Cu(II) one—50 ppm.

To confirm the flocculating ability of AN PAM with the concentration of 50 ppm, the CPS analysis of the C-SiO_2_ suspension was performed. The results obtained for AN PAM-12% are presented in Figure 7. The data noted for AN PAM-40% were analogous. 

The CPS analysis confirmed that anionic polyacrylamide (50 ppm) had flocculating ability relative to the C-SiO_2_ particles. In the system containing no adsorbate, the most numerous particles were characterized by diameter of 0.44 μm. In the presence of AN PAM-12%, their size equaled 0.56 μm, whereas in the mixed AN PAM-12%/Cu(II) system this parameter was 1.04 μm. Based on these results, it can be stated that copper(II) ions enhanced the C-SiO_2_ aggregation occurring in the anionic polymer presence.

## 4. Discussion

The adsorption measurements showed that at pH 6 all types of polyacrylamide were adsorbed on the composite surface. However, the adsorbed amounts were significantly higher in the case of cationic polymers (compared to anionic ones). This phenomenon is mainly dictated by the character of electrostatic interactions occurring between the macromolecular compound and the solid. Anionic polyacrylamides contain carboxylic groups in their macromolecules. At pH 6 some of these moieties are dissociated and, as a result, AN PAM is negatively charged. On the other hand, cationic polyacrylamides contain quaternary amine groups. Under examined conditions, some of them are also dissociated and contribute to positive charge of CT PAM. As previously mentioned, at pH 6 the composite surface is negatively charged (σ_0_ = -8.01 µC/cm^2^; ζ = -9.21 mV). Thus, there is an electrostatic attraction between positively charged CT PAM macromolecules and negative solid surface, which promotes the adsorption process. In turn, in the system containing anionic polymer, there is an electrostatic repulsion between negative AN PAM and negative solid particles, which significantly impedes the contact between system components. Owing to above interactions, the AN PAM adsorbed amounts are clearly smaller than those of CT PAM.

Among cationic polyacrylamides, higher adsorbed amount was observed for CT PAM-25%. At pH 6, the dissociation degree (α) of cationic polymers is close to 1, which means that practically all of the quaternary amino groups in their macromolecules are dissociated. CT PAM-25% contains a larger number of positive groups and therefore the electrostatic attraction between its macromolecules and the solid is stronger than in the case of CT PAM-15%. Among anionic polyacrylamides, a larger adsorbed amount was also noted for the polymer containing greater number of ionizable functional groups (AN PAM-40%). This observation is primarily dictated by the different conformation of AN PAM-12% and AN PAM-40% macromolecules on the composite surface. At pH 6, the dissociation degree of AN PAM-12% is 0.98, while of AN PAM-40%—0.83. This means that under selected conditions, AN PAM-12% contains about 11.7% of dissociated carboxylic groups, whereas AN PAM-40%—about 33.2%. Due to this fact, AN PAM-40% containing greater number of dissociated functional groups are characterized by more expanded conformation in the solution. Owing to strong electrostatic repulsion between polymer chains and the solid, AN PAM-40% creates long structures like "loops" and "tails" on the composite surface. Within this conformation, the number of contact sites of AN PAM-40% and C-SiO_2_ is limited. A single macromolecule takes a relatively small part of the solid and therefore large number of AN PAM-40% chains can be adsorbed on unit solid surface. The smaller number of dissociated carboxylic groups in AN PAM-12% contributes to its more coiled conformation in the solution as well as in the adsorption layer. A single macromolecule occupies a large part of the surface and thus smaller number of AN PAM-12% can interact with C-SiO_2_. This makes the AN PAM-12% adsorption lower. Under electrostatic repulsion between adsorbent and adsorbate, polymer adsorption on the composite surface is mainly caused by the formation of hydrogen bonds [41,42].

Wiśniewska et al. also examined the impact of functional group content on the polyacrylamide adsorbed amount. This team used montmorillonite [42], alumina [43] and chromium(III) oxide [44] as adsorbents in the studies. It was found that on the montmorillonite surface polyacrylamide containing greater number of carboxylic groups was adsorbed in smaller quantity. In turn, in the case of alumina and chromium(III) oxide, the adsorbed amounts of polyacrylamide with a larger number of carboxylic groups were higher. Fijałkowska et al. [22] observed a higher adsorption level of polyacrylamide containing a greater number of quaternary amine groups on the montmorillonite surface. Other researchers also examined the adsorption process on the solid surface, e.g., on magnesium hydroxide particles [45] and talc [46].

The performed experiments also indicated that the carbon–silica composite adsorbed a certain amount of copper(II) ions. The highest Cu(II) adsorbed amount observed in the examined system was equal to 9.8 mg/g. Other researchers also examined copper(II) adsorption on various solids. Jin et al. [47] found that algae–dairy–manure slurry pyrolyzed at 400 °C adsorbed 21.12 mg/g Cu^2+^ at pH 6 in 1440 min. Shim et al. [48] observed that giant *Miscanthus* pyrolyzed at 600 °C adsorbed 15.4 mg/g Cu^2+^ at pH 6 in 2880 min. In turn, Hoslett et al. [49] showed that biochar derived from the pyrolysis of municipal solid waste adsorbed 3.82 mg/g Cu^2+^ at pH 6 in 120 min. In the previous study, our team found that at pH 6 hay-based activated biochar obtained by chemical activation in a microwave furnace adsorbed 68.2 mg/g [30].

The Cu(II) ions added to the system significantly affected the adsorbed amount of ionic polyacrylamide. Heavy metal ions increased the adsorbed amounts of AN PAM and reduced those of CT PAM. In the system containing Cu(II) ions and anionic polymer, the formation of Cu(II)-AN PAM complexes may occur because there is an attraction between metal cations and negatively charged macromolecules. The created complexes are characterized by less negative net charge than AN PAM chains and thus their adsorption is more favorable. On the other hand, cationic polyacrylamide and Cu(II) ions compete with each other for access to the C-SiO_2_ surface. As a result, the polymer adsorption is limited. The same mechanisms and phenomena are responsible for higher Cu(II) adsorbed amounts in the presence of AN PAM and lower Cu(II) adsorbed amounts in the presence of CT PAM (in comparison to the systems without polymers).

The impact of anionic polyacrylamide on the C-SiO_2_ aggregation is dependent on the polymer concentration. When it is equal to 50 ppm, AN PAM stimulates the aggregation of all particles. Large sedimenting aggregates, confirmed by CPS analysis, are formed and thus the system is clarified. This phenomenon is strongly connected with flocculation, which is based on the creation of polymer bridges between the solid particles. This process occurs mainly when the solid surface is not completely covered with the polymer, i.e., for low polymer adsorbed amounts on the solid. Moreover, it is possible even when zeta potential values of the solid particles are negative [50,51]. When the AN PAM concentration is 100 ppm, the macromolecular compound contributes to the aggregation of only part of particles. Some of them are still suspended in the solution and give the system relatively high turbidity. Under these conditions, due to larger surface coverage with the polymer, a clear flocculation is not possible. There are electrosteric forces between solid particles that improve the system stability [52]. These interactions are based on electrostatic repulsion between negative groups of adsorbed macromolecules as well as steric repulsion between adsorption layers. The same phenomena occur in the systems containing 50 and 100 ppm of CT PAM. For both initial concentrations of cationic polyacrylamides, the adsorbed amounts are relatively high, which prevents flocculation. Flocculating ability of polyacrylamide at appropriate concentration was also confirmed by other researchers [42,43].

Cu(II) ions minimally affect the C-SiO_2_ aggregation, which is probably dictated by their low adsorbed amount on the solid surface. When the Cu(II) ions are added to the system with the polymer, they do not change the polymer effect on the solid aggregation. In other words, the selected macromolecular compounds retain their abilities to stabilize or destabilize the suspension in the presence of heavy metal. It must be also mentioned that in the mixed system of AN PAM (50 ppm) and Cu(II), heavy metal ions enhance the C-SiO_2_ aggregation. Then the AN PAM-Cu(II) complexes of slight net charge are adsorbed on the surface and, as a result, the particles can easily aggregate.

## 5. Conclusions

The preformed study allowed for the following conclusions. An innovative carbon–silica composite may be used as an adsorbent during water purification or wastewater treatment. Moreover, it can be applied as soil additive immobilizing copper(II) ions. This solid adsorbs 9.8 mg/g when the heavy metal initial concentration is 100 ppm. When the anionic polyacrylamide is present in the system, the Cu(II) adsorbed amount on Ci-SiO_2_ is higher and equals 16 mg/g. However, cationic polyacrylamide limits the adsorption ability of the examined composite. When CT PAM-25% is added to the system, the Cu(II) adsorbed amount is 1.5 mg/g. 

The examined composite adsorbs ionic polyacrylamide macromolecules. Due to more favorable electrostatic interactions, cationic polyacrylamide adsorbed amounts on the composite are higher than those of anionic one. The adsorbed amount of AN PAM-12% is 3.45 mg/g, of AN PAM-40%—4.62 mg/g, of CT PAM-15%—25.46 mg/g, whereas of CT PAM-25%—35.72 mg/g. Cu(II) ions enhance the adsorption of anionic polymers. Then the adsorption efficiency of AN PAM is almost 100% (49.83 mg/g for AN PAM-40%). In turn, the adsorbed amounts of cationic polymers in the presence of heavy metal ions are lower. For example, for CT PAM-15%, this parameter equals 21.08 mg/g. 

The C-SiO_2_ aggregation is dependent on both type and concentration of polyacrylamide. Flocculating ability is observed only in the case of anionic polyacrylamides with the concentration of 50 ppm. Then, the flocculation process is possible. Cu(II) ions, added to this system, stimulate the solid aggregation additionally. These data may be very helpful in the development of procedures of solid separation from aqueous media as well as during soil remediation.
